# Correction for Hirose et al., “Impact of a Demyelination-Inducing Central Nervous System Virus on Expression of Demyelination Genes in Type 2 Lymphoid Cells”

**DOI:** 10.1128/JVI.00209-21

**Published:** 2021-04-12

**Authors:** Satoshi Hirose, Mihoko Kato, Kati Tormanen, Pedram Shafiei Jahani, Omid Akbari, Homayon Ghiasi

**Affiliations:** a Center for Neurobiology & Vaccine Development, Ophthalmology Research, Department of Surgery, Cedars-Sinai Medical Center, Los Angeles, California, USA; b Department of Biology, Pomona College, Claremont, California, USA; c Department of Molecular Microbiology and Immunology, Keck School of Medicine, University of Southern California, Los Angeles, California, USA

## AUTHOR CORRECTION

Volume 95, no. 4, e01934-20, 2021, https://doi.org/10.1128/JVI.01934-20. The article was originally published on 28 January 2021 with a standard copyright line (“© 2021 American Society for Microbiology. All Rights Reserved.”). We elected to pay for open access for the article after publication, necessitating replacement of the original copyright line with “© 2021 Hirose et al. This is an open-access article distributed under the terms of the Creative Commons Attribution 4.0 International license.” This change was made to the online version of the article on 17 February 2021.

